# Electroacupuncture Restores Locomotor Functions After Mouse Spinal Cord Injury in Correlation With Reduction of PTEN and p53 Expression

**DOI:** 10.3389/fnmol.2018.00411

**Published:** 2018-11-16

**Authors:** Zhe Wei, Weijiang Zhao, Melitta Schachner

**Affiliations:** ^1^Center for Neuroscience, Shantou University Medical College, Shantou, China; ^2^Faculty of Medicine and Health, Lishui University, Lishui, China; ^3^Department of Cell Biology and Neuroscience, Rutgers University, Piscataway, NJ, United States

**Keywords:** electroacupuncture, spinal cord injury, p53, PTEN, mTOR, mouse

## Abstract

**Background**: We previously showed that electroacupuncture (EA) at Jiaji points promotes expression of adhesion molecule L1 in spinal cord tissue after mouse spinal cord injury (SCI) and contributes to recovery of neural functions.

**Objective**: We investigated the effects of EA on downstream signaling molecules of L1 and molecules relevant to apoptosis with the aim to understand the underlying molecular mechanisms.

**Methods**: Female C57BL/6 mice were divided into a sham group, injury group, injury+acupuncture (AP) group and injury+EA group. We investigated the changes in cognate L1-triggered signaling molecules after SCI by immunofluorescence staining and immunoblot analysis.

**Results**: Protein levels of phosphatase and tensin homolog (PTEN) and p53 were decreased by EA at different time points after injury, whereas the levels of phosphorylated mammalian target of rapamycin (pmTOR), p-Akt and phosphorylated extracellular signal-regulatedkinase (p-Erk) were increased. Also, levels of myelin basic protein (MBP) were increased by EA. AP alone showed less pronounced changes in expression of the investigated molecules, when compared to EA.

**Conclusion**: We propose that EA contributes to neuroprotection by inhibiting PTEN and p53 expression and by increasing the levels of pmTOR/Akt/Erk and of MBP after SCI. These observations allow novel insights into the beneficial effects of EA via L1-triggered signaling molecules after injury.

## Introduction

Spinal cord injury (SCI) results in a series of pathophysiological changes, such as limiting regrowth/sprouting of severed axons, increasing apoptosis, and influencing pro- and anti-inflammatory reactions, all impinging on the devastating paralysis resulting from the lesion (Chen et al., 2017; Shen et al., 2017). Recognition molecules, cytokines, and neurotrophins all contribute in various ways to enhancing or reducing recovery. Apoptosis has been suggested to be an important player in affecting recovery after SCI. Intrinsic and extrinsic factors trigger apoptosis, activating, among others, caspases and nucleases, eventually leading to cell damage (Nicola et al., 2017; Shultz et al., 2017). The progression of apoptosis is influenced by a diverse range of pathways, among which PI3K/Akt/mammalian target of rapamycin (mTOR) is one of the well-known ones (Bao et al., 2016; Chen C.-H. et al., 2016; Gao et al., 2016; Wang et al., 2017; Zhao H. et al., 2017). Preventing apoptosis is an important aim in ameliorating the consequences inflicted by SCI.

Acupuncture (AP) has been used worldwide in clinical treatment because of its beneficial therapeutic effects. Studies have been carried out to gain insights into the molecular mechanisms underlying these benefits. Electroacupuncture (EA) combines AP with a continuous low frequency current which has proven to be more effective than AP alone by supporting survival of the lesioned tissue by carefully dosed electrical activation (Lin et al., 2015; Escobar-Corona et al., 2017). Previous studies indicated that EA not only improves locomotor function after SCI as assayed by behavioral studies (Wei et al., 2017), but also enhances expression of trophic molecules (Mo et al., 2016; Fang et al., 2017; Min et al., 2017; Wei et al., 2017; Zhao J. et al., 2017). The mechanisms underlying the beneficial outcome following EA treatment are complex, involving cytokines, neurotrophins, epigenetic influences, and the immune system, to name only a few (Liu et al., 2014; Renfu et al., 2014; Ding et al., 2015; Geng et al., 2015). EA at acupoints in the “Governer Vessel” has been reported to inhibit the Notch signaling pathway and to enhance the phosphorylation of mTOR/Akt/extracellular signal-regulated kinase (Erk; Lee et al., 2014; Ohtake et al., 2014). A negative regulator of recovery after injury is phosphatase and tensin homolog (PTEN; Walker et al., 2013; Danilov and Steward, 2015), which can inhibit neuritogenesis and axonal regrowth after injury (He et al., 2012). PTEN reduces the generation of PIP3 from PIP2, while the PI3K pathway is activated in the absence of PTEN. PIP3 recruits cellular proteins containing lipid-binding domains to cell membrane and binds to the pleckstrin homology domain in the N-terminal region of Akt. mTOR is a signaling molecule downstream of Akt leading to control and coordination of neural and muscle regrowth after SCI (Chen N.-N. et al., 2016; Zhou et al., 2016; Bai et al., 2017). Notwithstanding its role in malignancy, the tumor suppressor protein p53 is important for nerve cell differentiation and axonal growth regrowth (Eom et al., 2015; Ma et al., 2017). Interestingly, under stress, activation of p53 can lead to shortening of neurites and decrease of neuronal activity (Guo et al., 2014; Eom et al., 2015; Ma et al., 2017). Another important factor in regeneration after trauma is myelin basic protein (MBP) which is an important contributor to formation and maintenance of myelin sheaths and thereby enhances functional recovery after injury. Furthermore, studies *in vitro* and *in vivo* have implicated MBP and MBP-mediated cleavage of L1 in recovery and regeneration after acute nervous system injury in adult mammals (He et al., 2012; Lutz et al., 2014, 2016; Xu et al., 2017).

Based on these findings, we analyzed in the present study the relationship between EA and the expression of PTEN and p53 after SCI in mice. We also investigated some molecular underpinnings of EA, namely phosphorylation of Akt, Erk, and mTOR, which are the cognate downstream signaling molecules that are triggered by L1. Lastly, it was important to measure expression of MBP, since MBP is an indicator of the structural status of myelin and serves to form and maintain mature myelin, without which axonal action potentials cannot be generated, thereby compromising essential nervous system functions.

## Materials and Methods

### Animals and Experimental Groups

Animals were obtained from the central animal facility of Shantou University Medical College and maintained in this facility. Female C57BL/6 mice, 10–12 weeks old, were used. Male mice were not studied because of the difficulties in handling these animals under SCI-induced stress and in manually voiding their bladders. Mice were maintained at 23 ± 2°C and 60 ± 10% humidity under a 12-h light/ dark cycle with *ad libitum* access to water and food. All experiments were performed in accordance with the governmental laws on the protection of experimental animals, as approved by the responsible committee of the State of Guangdong (Permit Number: SUMC2015-041).

Mice were divided into four groups: sham group, injury group, injury+AP group and injury+EA group, with 40 mice in each group. The sham group received only laminectomy at the T9–T11 levels. The other two groups underwent SCI at the T9–T11 segments of the spinal cord as follows: the injury group received only SCI; the injury+AP group received AP treatment, starting 1 day after SCI; the injury+EA group received electronically driven AP treatment, starting 1 day after SCI.

### Spinal Cord Injury

C57BL/6 mice, 10–12 weeks old, were obtained from the central animal facility of the Shantou University Medical College and were maintained in this facility under a 12-h light/dark cycle with *ad libitum* access to water and food. All experiments were performed in accordance with the governmental and international laws on the protection of experimental animals, as approved by the committee of the State of Guangdong (Permit Number: SUMC2015-041).

Mice were randomly divided into four groups: sham group, injury group, injury+AP group and injury+EA group. Forty mice were randomly assigned to each group. The sham group received only a laminectomy at the T9–T11 levels. The other three groups underwent SCI at the T9–T11 segments of the spinal cord as follows: the injury group received only SCI; the injury+AP group received AP treatment on the next day after SCI; and the injury+EA group received electronically driven AP treatment starting on the next day after SCI (Wei et al., 2017).

All SCI surgeries were performed under aseptic conditions as described (Pan et al., 2014; McDonough et al., 2015; Wei et al., 2017). Mice were anesthetized by intraperitoneal injections with a mixture of ketamine (60 mg/kg, Fujian Gutian Pharmaceutical, Ningde, Fujian, China) and xylazine (5 mg/kg, Sigma-Aldrich). The skin on the back of mice was shaved and disinfected, and the dorsal aspect of the spinal column was exposed. Eyes were protected by an ointment (Chongqing Kerui Pharmaceutical, Nanping, Chongqing, China). A laminectomy was carried out at the T9–T11 levels to expose the spinal segment without damaging the dura or touching adjacent skin segments. The spinal cord was severely compressed for 15 s, using a pair of forceps (Fine Science Tools, Heidelberg, Germany) under standardized conditions using a calibrated electromagnetic device (Pan et al., 2014). The extent of the injury was evident by the animals’ inability to move their hind legs which were dragged behind. Injury completeness was always checked before proceeding for the AP experiments. After injury, the mice were placed into a humidity-controlled chamber overnight at 28°C. They were then housed singly with water and food *ad libitum*. The bladders were emptied manually two times per day until reflexive bladder emptying had normalized. The sham group only received a laminectomy, but no injury to the spinal cord, and was treated identically to the injury groups.

### Acupuncture and Electroacupuncture

Jiaji points were chosen for EA treatment (Wei et al., 2017). There are 17 pairs of Jiaji points bilaterally at the levels of the cervix, thorax and waist (Wei et al., 2017). Mice were kept without anesthesia in an immobilization apparatus. Two pairs of stainless steel needles (0.25 × 13, 0.25 mm in diameter, Zhongyan Taihe Medical Machine Company, Beijing, China) were inserted into the Jiaji points in the thorax, rostral and caudal to the injury center at the T7 and T11 levels. In the injury+EA group, the needles were connected to the output terminals of a KWD808 I electronic AP device (Guangzhou Yingdi Electronic Medical Device Co. Ltd., Guangzhou, China) and stimulated by a dense-disperse wave of 2/100 Hz frequency and 0.2 mA intensity for 15 min, calibrated as described (Wei et al., 2017). For the AP group, the needles were swiftly twisted manually at a rate of two spins per second for 10 s every 5 min during a 15-min period. In the AP group, mice only received AP but no electric stimulation. EA and AP were started 1 day after surgery and continued for 5 days, being followed by 1 day of rest. Thereafter, EA and AP were continued to be performed every day with 1 day of rest in the weekly treatment, lasting for 28 days after SCI. The success of the AP and EA treatments was robust from experiment to experiment as evidenced by the small errors of deviation throughout the trials.

### Behavioral Analyses

Two methods were used to assess the outcome of treatments. BMS locomotor analysis, including BMS score and BMS subscore tests (Salewski et al., 2015; Wei et al., 2017) was used to analyze locomotor behavior at 1, 3, 7, 14, and 28 days after SCI by two experienced persons blinded to the treatment. Individual BMS scores from each animal ranged from 0 (no ankle movement) to 9 (entire functional recovery) points, and 11 points for the BMS subscore. The correlations between BMS score and PTEN level at 7 days after injury and between BMS score and pmTOR level at 28 days were analyzed.

### Immunohistology

Immunohistology was performed as described (McDonough et al., 2015; Sauce et al., 2015). At different time points (3, 7, 14 and 28 days) after SCI, mice were anesthetized with isopentane (Baxter Healthcare Puerto Rico, Guayama, Puerto Rico) by inhalation and perfused via cardiac puncture first with PBS and then with 4% formaldehyde in PBS (Tang et al., 2015; Wang et al., 2015). Antigen de-masking was also performed as described in 0.01 M sodium citrate solution (pH 6.0) at 99°C for 40 min (Han et al., 2013; Chen et al., 2015). After washing the sections in PBS three times, 5 min each, at room temperature (RT), they were blocked for 1 h at RT using 5% normal donkey serum in PBS containing 0.2% Triton X-100. Incubation with polyclonal antibody against phosphorylated mTOR (pmTOR, 1:500, # sc-293133, Santa Cruz Biotechnology) was carried out overnight at 4°C. After washing three times with PBS (3 × 5 min at RT), sections were incubated with secondary antibodies: Alexa Fluor 594 goat anti-mouse (1:1,000, # A31623, Life Technologies) for 1 h at RT, then washed with PBS (3 × 5 min at RT). The sections were then incubated for 10 min at RT with the nuclear marker DAPI (1:5,000, # C1002, Beyotime Institute of Biotechnology, Shanghai, China) and mounted on coverslips (Citotest Labware Manufacturing Company, Jiangsu, China). Photographic documentation was performed with an Axiophot 2 microscope equipped using a digital AxioCamHRc camera and AxioVision software (Zeiss, Oberkochen, Germany). For quantitative evaluation, images were processed using ImageJ software as described in the manual.

### Immunoblot Analysis

Immunoblot analysis was performed as described (Chen et al., 2015; Wang et al., 2015) to measure levels of PTEN, p53, pmTOR, pErk1/2, pAkt and MBP. Spinal cords were dissected out on ice with the injury site at the center and 0.5 cm tissue each rostral and caudal to the injury site. Isolated tissue was homogenized using a Dounce homogenizer and then centrifuged at 12,000 *g* and 4°C for 15 min. Supernatant samples (20 μg) were separated by SDS-PAGE and transferred to a nitrocellulose membrane (Santa Cruz Biotechnology) which was blocked in 1% non-fat skim milk or 0.3% bovine serum albumin in PBS containing 0.01% Tween-20 (TBST) for 1 h at RT. The membranes were then incubated with mouse monoclonal anti-pmTOR (diluted 1:1,000; # sc-293133, Santa Cruz Biotechnology), rabbit polyclonal anti-PTEN (diluted 1:1,000; # sc-7974, Santa Cruz Biotechnology), mouse monoclonal anti-p53 (diluted 1:1,000; # sc-47698, Santa Cruz Biotechnology), mouse monoclonal anti-pErk1/2 (diluted 1:1,000; # sc-514302, Santa Cruz Biotechnology), rabbit polyclonal anti-MBP (diluted 1:1,000; # sc-808, Santa Cruz Biotechnology), rabbit polyclonal anti-Akt (diluted 1:1,000; # sc-348400, Life Technologies), mouse monoclonal anti-GAPDH (diluted 1:5,000; # sc-47724, Santa Cruz Biotechnology) or mouse monoclonal anti-actin (diluted 1:1,000; # sc-8432, Santa Cruz Biotechnology) at 4°C overnight. After washing with TBST (3 × 5 min at RT), membranes were incubated with the appropriate secondary antibodies (Jackson Immunoresearch) for 1 h, and immunoreactive bands were visualized by chemiluminescence using Supersignal (Bio-Rad Laboratories). GAPDH or actin was used as loading controls. Relative intensity of bands was analyzed by ImageJ software as described in the manual.

### Statistical Analysis

The statistical package SPSS software, version 19.0 (SPSS, Inc., Chicago, IL, USA) was used for all analyses. Data were analyzed by one-way analysis of variance (ANOVA), and comparisons between groups were performed using Tukey’s *post hoc* test. Correlation assay was performed by the Pearson correlation coefficient analysis. All values are expressed as means ± SEM. *P* < 0.05 and *p* < 0.01 were both considered to indicate a statistically significant difference.

## Results

### AP and EA Increase p-Akt Levels After SCI

**Figure 1:**
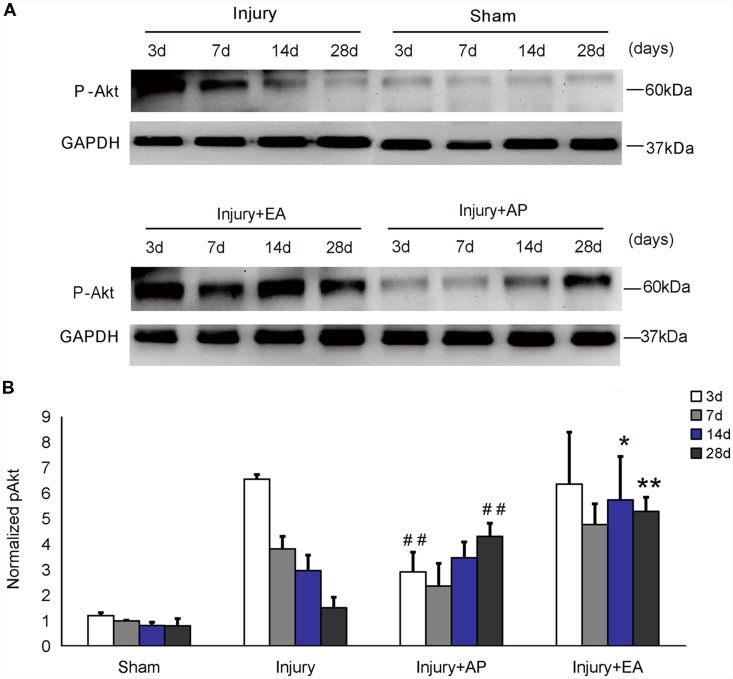
Electroacupuncture (EA) increases phosphorylation of Akt at 14 and 28 days after spinal cord injury (SCI). Acupuncture (AP) increases phosphorylation of Akt at 28 days after SCI. Spinal cord tissues were prepared and assessed by immunoblot analysis at the indicated days. **(A)** Representative immunoblots for p-Akt and GAPDH. The intensity of bands was measured by ImageJ software. **(B)** p-Akt normalized to GAPDH. *,**Denote differences between injury+EA group and the injury groups in p-Akt levels at 14 and 28 days after SCI (*p* < 0.05, *p* < 0.01, respectively). ^##^Denotes differences between the injury+AP group and the injury groups in p-Akt levels at 3 and 28 days after SCI (*p* < 0.01). Data are presented as means ± SEM (four mice/group in three independent experiments, *p < 0.05, **^/##^*p* < 0.01, one-way analysis of variance (ANOVA), Tukey’s *post hoc* test).

To investigate whether AP and EA affect phosphorylation levels of Akt, we performed immunoblot analysis at 3, 7, 14 and 28 days after SCI on spinal cord tissue containing the injury site at the center and 0.5 cm tissue each rostral and caudal to the injury site (Figure 1). In the injury group, p-Akt decreased gradually after SCI. p-Akt was enhanced in the injury+EA group after 7 days and was higher compared to the injury group at 14 and 28 days (means ± SEM are 5.7415 ± 2.90 and 2.9715 ± 0.3697, *t* = 2.6533, *p* = 0.028; means ± SEM are 5.2961 ± 0.30 and 1.5092 ± 0.1737, *t* = 9.5275, *p* = 0.0003, respectively). Results indicate that EA promotes p-Akt levels at both 14 and 28 days after SCI, whereas AP enhances p-Akt levels only at 28 days (means ± SEM are 4.3154 ± 0.268 and 1.5092 ± 0.1737, *t* = 7.3125, *p* = 0.0009). We conclude that EA enhances phosphorylation of Akt more readily than AP.

### AP and EA Increase of p-Erk Levels After SCI

**Figure 2:**
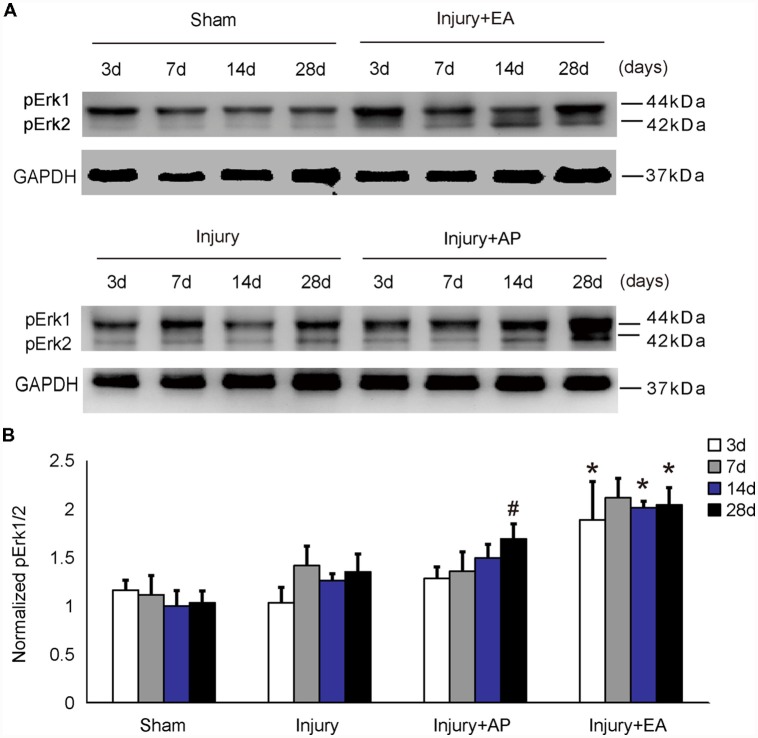
EA increases phosphorylated extracellular signal-regulated kinase 1/2 (p-Erk1/2) levels at 3, 14 and 28 days after SCI. AP increases p-Erk1/2 levels at 28 days after SCI. Spinal cord tissues were prepared and assessed by immunoblot analysis at the indicated days. **(A)** Representative immunoblots for p-Erk and GAPDH. The intensity of bands was measured by ImageJ software. **(B)** p-Erk normalized to GAPDH. ^#^Denotes differences between the injury+AP group and the injury groups in p-Akt levels at 28 days after SCI (*p* < 0.05). *Denotes differences between the injury+EA group and the injury groups in p-Akt levels at 14 and 28 days after SCI (*p* < 0.05). Data are presented as means ± SEM (four mice/group in three independent experiments, *^/#^*p* < 0.05, one-way ANOVA, Tukey’s *post hoc* test).

To investigate whether AP and EA affect p-Erk levels, we performed immunoblot analysis at 3, 7, 14 and 28 days after SCI on spinal cord tissue (Figure 2). p-Erk levels in the injury+AP group were higher than in the injury group at 28 days (means ± SEM are 2.8793 ± 1.1559 and 1.2274 ± 0.3533, *t* = 2.3289, *p* = 0.0401). Even higher levels of p-Erk were observed in the injury+EA group at 3, 14 and 28 days of SCI (means ± SEM are 2.4361 ± 0.2559 and 1.3052 ± 0.4157, *t* = 2.39, *p* = 0.0375, means ± SEM are 2.8793 ± 1.156 and 1.0896 ± 0.2245, *t* = 2.6382, *p* = 0.0201, and means ± SEM are 1.8894 ± 0.2347 and 1.0325 ± 0.0375, *t* = 2.8451, *p* = 0.0239, respectively). These results indicate that EA promotes p-Erk levels at 3, 14 and 28 days, whereas AP enhances p-Erk levels only at 28 days after SCI.

### AP and EA Increase pmTOR Levels After SCI

**Figure 3:**
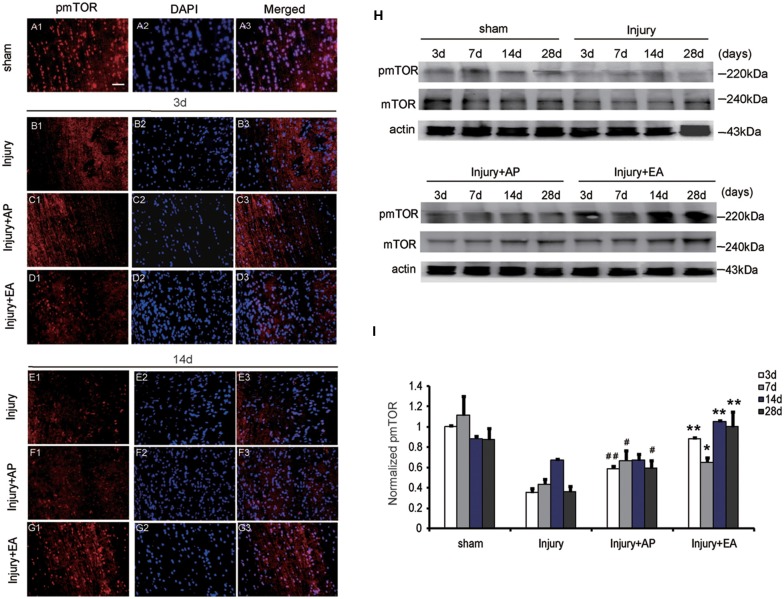
EA increases phosphorylated mammalian target of rapamycin (pmTOR) levels at 3, 7, 14 and 28 days after SCI. AP increases pmTOR levels at 3, 7 and 28 days after SCI. Spinal cord tissues were prepared and assessed by immunofluorescence and immunoblot analyses at the indicated days. **(A1–G3)** Representative micrographs of pmTOR and DAPI staining at 3 days and 14 days after SCI in different groups. Scale bar = 20 μm. **(H)** Representative immunoblots for pmTOR, mTOR and actin. The intensity of bands was measured by ImageJ software. **(I)** Relative expression of pmTOR. ^##^,^#^Denote differences between the injury+AP group and the injury groups in pmTOR levels at 3, 7 and 28 days after SCI (*p* < 0.01, *p* < 0.05). **,*Denote differences between injury+EA group and the injury groups in expression of phosphatase and tensin homolog (PTEN) at 3, 7, 14 and 28 days after SCI (*p* < 0.01, *p* < 0.05). Data are presented as means ± SEM (four mice/group in three independent experiments, *^/#^*p* < 0.05, **^/##^*p* < 0.01, one-way ANOVA, Tukey’s *post hoc* test).

To evaluate whether AP and EA affect the pmTOR after SCI, we performed immunofluorescence staining and immunoblot analyses. Spinal cord tissue was dissected out for immunoblot analysis and, for immunohistology, longitudinal sections were taken 1 cm caudal to the lesion site at 3 and 14 days after SCI (Figures 3A1–G3, showing representative micrographs of pmTOR and DAPI stainings at 3 and 14 days after SCI in the different groups. Figures 3A1–A3 show levels of pmTOR in the sham group. At 3 days after SCI, levels of pmTOR were decreased compared with the other groups: Figures 3B1–B3 representing the injury group without AP; Figures 3C1–C3 representing the injury+AP group; Figures 3D1–D3 representing the injury+EA group). Compared with the injury group, levels of pmTOR were increased in the injury+AP group and the injury+EA group at 3 days after SCI (means ± SEM are 0.5882 ± 0.0004 and 0.3552 ± 0.0013, *t* = 9.5457, *p* = 0.0003; means ± SEM are 0.8804 ± 0.0001 and 0.3552 ± 0.0013, *t* = 12.6282, *p* = 0.0001, respectively). At 7 days after SCI, pmTOR levels were enhanced in the injury+AP and injury+EA groups when compared to the injury group (means ± SEM are 0.6684 ± 0.014 and 0.4338 ± 0.0026, *t* = 3.1771, *p* = 0.0168; means ± SEM are 0.6535 ± 0.0069 and 0.4338 ± 0.0026, *t* = 3.8956, *p* = 0.0088, respectively). Levels of pmTOR were higher in the injury+AP group than in the injury group at 28 days (means ± SEM are 0.5936 ± 0.0053 and 0.3623 ± 0.0031, *t* = 4.3551, *p* = 0.0061). An even higher increase was observed in the injury+EA group in comparison with the injury group at 14 and 28 days (means ± SEM are 1.0869 ± 0.0001 and 0.6715 ± 0.0001, *t* = 14.4493, *p* = 0.0001; means ± SEM are 1.0462 ± 0.0203 and 0.3623 ± 0.0031, *t* = 7.7337, *p* = 0.0008, respectively; Figures 3H,I), indicating that both treatments lead to enhanced mTOR phosphorylation after SCI, with EA being more efficient than AP.

### AP and EA Reduce PTEN Levels After SCI

**Figure 4:**
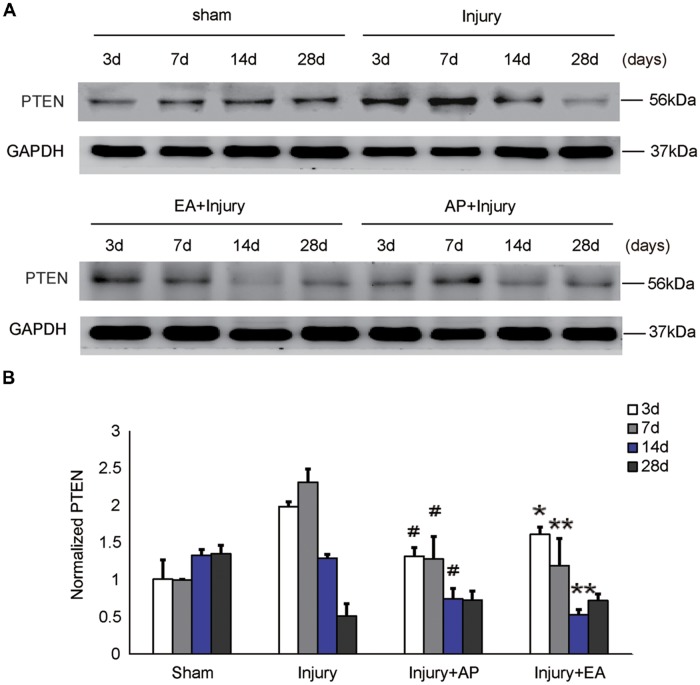
AP and EA inhibit the expression of PTEN at 3, 7 and 14 days after SCI. Spinal cord tissue was assessed by immunoblot analysis at the indicated days. **(A)** Representative immunoblots for PTEN and GAPDH. **(B)** PTEN expression levels normalized to GAPDH. ^ #^Denotes differences between the injury+AP group and the injury groups in expression of PTEN at 3, 7 and 14 days after SCI (*p* < 0.05). *,**Denote differences between injury+EA group and the injury groups in expression of PTEN at 3, 7 and 14 days after SCI (*p* < 0.05, *p* < 0.01). Data are presented as means ± SEM (four mice/group in three independent experiments, *^/#^*p* < 0.05, **p < 0.01, one-way ANOVA, Tukey’s *post hoc* test).

To investigate whether AP and EA affect the expression of PTEN, we performed immunoblot analysis at 3, 7, 14 and 28 days after SCI (Figure 4). Levels of PTEN were increased at 3 and 7 days and then decreased after 14 days in the injury group. Levels of PTEN were decreased in the injury+AP group at 3, 7 and 14 days after SCI (means ± SEM are 1.3874 ± 0.1853 and 2.0539 ± 0.3931, *t* = 2.7891, *p* = 0.0258; means ± SEM are 1.2936 ± 0.0899 and 2.3963 ± 0.3581, *t* = 4.8422, *p* = 0.0006; means ± SEM are 0.7986 ± 0.0593 and 1.2975 ± 0.4331, *t* = 2.5979, *p* = 0.0301, respectively). In the injury+EA group, PTEN expression was also lower compared to the injury group and more sustained at lower levels, compared to the injury group, at 7 and 14 days after SCI (means ± SEM are 1.1455 ± 0.2943 and 2.3963 ± 0.3581, *t* = 4.5996, *p* = 0.0005; means ± SEM are 0.5375 ± 1.643 and 1.2975 ± 0.4331, *t* = 2.2843, *p* = 0.0041, respectively). These results indicate that AP and, more strongly, EA reduce PTEN levels after SCI.

### AP and EA Reduce p53 Levels After SCI

**Figure 5:**
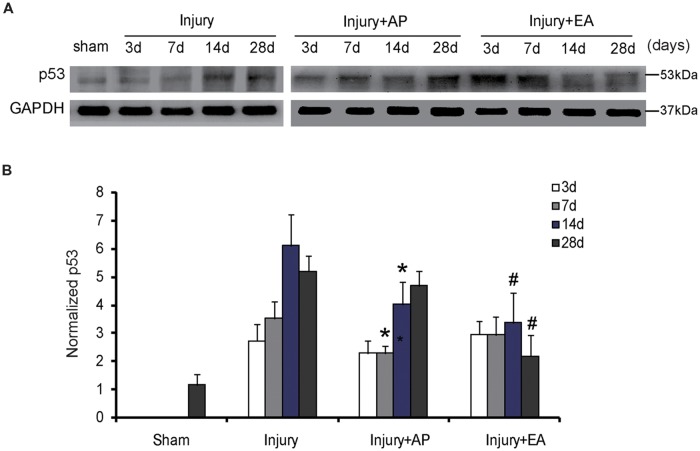
EA reduces expression of p53 at 14 and 28 days after SCI. AP reduces expression of p53 at 7 and 14 days after SCI. Spinal cord tissues were prepared and assessed by immunoblot analysis at the indicated days. **(A)** Representative immunoblots for p53 and GAPDH. The intensity of bands was measured by ImageJ software. **(B)** p53 normalized to GAPDH. *Denotes differences between the injury+AP group and the injury groups in expression of p53 at 7 and 14 days after SCI (*p* < 0.05). ^#^Denotes differences between the injury+EA group and the injury groups in expression of p53 at 14 and 28 days after SCI (*p* < 0.05). Data are presented as means ± SEM (four mice/group in three independent experiments, *^/#^*p* < 0.05, one-way ANOVA, Tukey’s *post hoc* test).

To investigate whether AP and EA affect p53 expression, we performed immunoblot analysis at 3, 7, 14 and 28 days after SCI (Figure 5). p53 levels were increased in the injury group at 3 days after SCI. Compared to the injury group, p53 expression was decreased in the injury+AP group at 7 and 14 days (means ± SEM are 2.1689 ± 1.4873 and 3.3578 ± 0.9745, *t* = 2.8305, *p* = 0.0281; means ± SEM are 4.0365 ± 2.0434 and 6.1935 ± 1.8345, *t* = 3.9461, *p* = 0.01573, respectively). In the injury+EA group levels of p53 were even more reduced in comparison to the injury group at 14 and 28 days after SCI (means ± SEM are 3.2945 ± 1.2423 and 6.1935 ± 1.8345, *t* = 3.8946, *p* = 0.01638; means ± SEM are 2.1353 ± 0.9384 and 5.1673 ± 1.5866, *t* = 3.8843, *p* = 0.01776, respectively). These results indicate that EA reduces p53 expression more effectively than injury+AP after SCI.

### AP and EA Increase Expression of MBP After SCI

**Figure 6:**
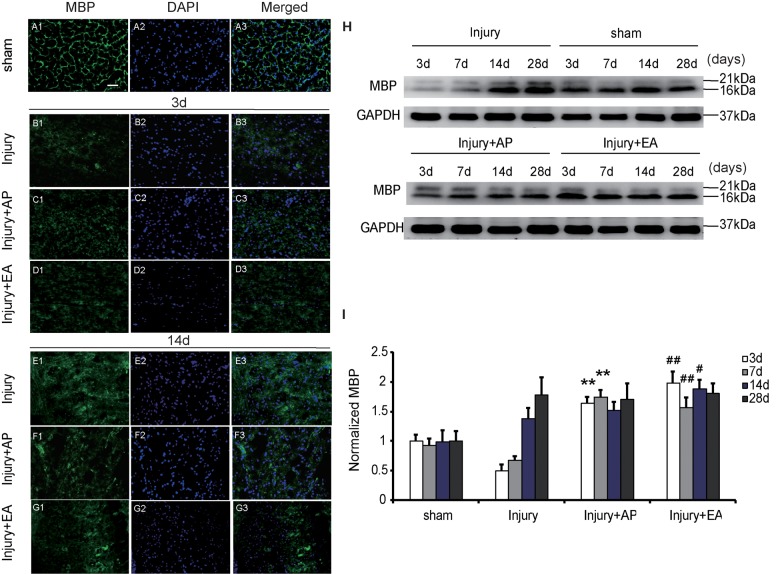
EA increases expression of myelin basic protein (MBP) at 3, 7 and 14 days after SCI. AP increases expression of MBP at 3 and 7 days after SCI. Spinal cord tissues were prepared and assessed by immunofluorescence and immunoblot analyses after SCI at the indicated days. **(A1–G3)** Representative images of MBP and DAPI staining at 3 and 14 days after SCI. Scale bar = 20 μm. **(H)** Representative immunoblots for MBP and GAPDH. **(I)** MBP normalized to GAPDH. **Denotes differences between the injury+AP group and the injury groups in expression of MBP at 3 and 7 days after SCI (*p* < 0.01). ^#^,^##^Denote differences between the injury+EA group and the injury groups in expression of MBP at 3, 7 and 14 days after SCI (*p* < 0.05, *p* < 0.01, respectively). Data are presented as means ± SEM (four mice/group in three independent experiments, ^#^*p* < 0.05, **^/##^*p* < 0.01, one-way ANOVA, Tukey’s *post hoc* test).

To evaluate whether AP and EA affect expression of MBP after SCI, we performed immunofluorescence staining and immunoblot analysis. The spinal cord tissue was harvested at 3 and 14 days after SCI (Figures 6A1–G3). Figures 6A1–G3 are representative images of MBP and DAPI staining at 3 and 14 days after SCI. Figures 6A1–A3 represent expression of MBP in the sham group, showing a normal structure. At 3 days, expression of MBP was decreased in the injury group compared with the other groups. Compared with the injury group, levels of MBP were increased in the injury+AP group and the injury+EA group at 3 days after SCI (means ± SEM are 1.6478 ± 0.0087 and 0.5028 ± 0.011, *t* = 12.1294, *p* = 0.0001; means ± SEM are 1.9909 ± 1.4873 and 0.5028 ± 0.011, *t* = 12.1179, *p* = 0.0001, respectively). At 7 days after SCI, expression of MBP was enhanced in the injury+AP group and the injury+EA group when compared to the injury group (means ± SEM are 2.1689 ± 1.4873 and 3.3578 ± 0.9745, *t* = 2.8305, *p* = 0.0006; means ± SEM are 2.1689 ± 1.4873 and 3.3578 ± 0.9745, *t* = 2.8305, *p* = 0.0001, respectively). At all time points studied, levels of MBP were higher in the injury+EA group than in the injury group (means ± SEM are 1.5672 ± 0.0288 and 0.6724 ± 0.0054, *t* = 8.3945, *p* = 0.0001; means ± SEM are 1.881 ± 0.0227 and 1.3791 ± 0.033, *t* = 3.68847, *p* = 0.01; Figures 6H,I). Our observations indicate that EA promotes expression of MBP at 3, 7 and 14 days, while AP improves MBP expression only at 3 and 7 days after SCI, showing a less sustainable effect.

### AP and EA Promote Locomotor Functions of Mice After SCI

**Figure 7:**
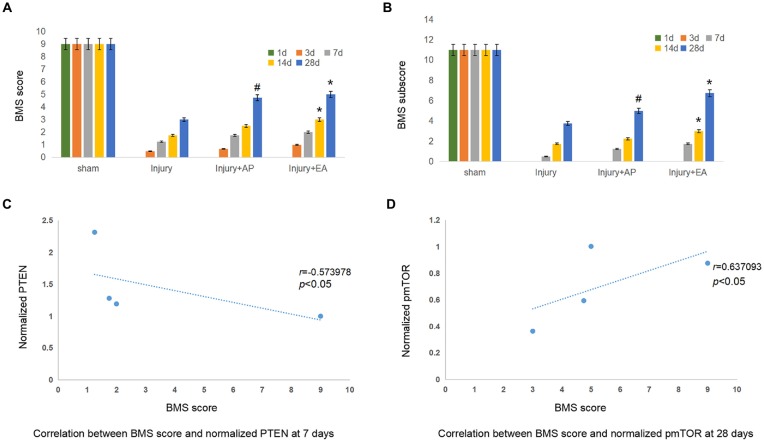
EA and AP improve locomotor recovery after mouse SCI as assessed by BMS analysis. BMS scores and BMS subscores are indicated at different days after SCI in reference to Wei et al. (2017). **(A)** BMS score. ^#^Denotes difference between injury+AP group and the injury group at 28 days after SCI (*p* < 0.05). *Denotes differences between the injury and injury+EA groups at 14 and 28 days after SCI (*p* < 0.05). **(B)** BMS subscore. ^#^Denotes difference between the injury+AP and the injury groups at 28 days after SCI (*p* < 0.05). *Denotes differences between injury+EA and the injury groups at 14 and 28 days after SCI (*p* < 0.05). **(C)** Correlation between BMS score and normalized PTEN at 7 days. (*r* = −0.573978). **(D)** Correlation between BMS score and normalized pmTOR at 28 days (*r* = 0.637093; pearson correlation coefficient analysis, *^/#^*p* < 0.05).

To determine the effect of EA and AP on locomotor function after SCI in the mouse, the BMS score and subscore were evaluated at 1, 3, 7, 14 and 28 days after SCI. BMS scores (Figure 7A) and BMS subscores (Figure 7B) were considerably reduced after SCI in the injury, Injury+AP and injury+EA groups, except for the sham group, which served as negative control. In the following days, the BMS score and BMS subscore gradually increased with time, especially at 7 days after SCI. AP improved the BMS score and BMS subscore compared with the injury group only at 28 days after SCI. Scores for the injury+EA group were higher than for the injury group at 14 and 28 days after SCI. The correlation coefficient assay results demonstrated that the BMS score was significantly correlated with normalized PTEN at 7 days (Figure 7C; *r* = −0.573978, *p* < 0.05). In addition, the BMS score was significantly correlated with normalized pmTOR at 28 days (Figure 7D; *r* = 0.637093, *p* < 0.05).

## Discussion

In the light of the traditional Chinese medicine theory of Zang-fu organs and meridians, the pathological basis of SCI involves “Governor Vessel” injury. The governor vessel plays an essential role in adjusting the overall blood circulation through the bladder meridian (Huang et al., 2015). Numerous studies have shown that EA at different Jiaji points exerts different effects in many diseases, as exemplified by observations on neurological problems in the central and peripheral nervous systems. Huatuo Jiaji points regulate the flow of body blood through the combined influence of the “Governor Vessel” and the bladder meridian. Also, EA at Jiaji points can produce a “Jiaji electric field” in the injured spinal cord segments after SCI.

Previous research in rats had indicated that EA at Jiaji points improves locomotor recovery and accelerates cortical somatosensory responses, which enhance functional recovery after SCI (Wu et al., 2012). In our previous studies, Catwalk Gait Analyses clearly showed that EA induced better locomotor recovery than AP, which in turn showed better recovery than the group of animals without AP as described (Wei et al., 2017). It has also been shown that EA activates tissue recovery in humans and other mammals, most likely by influencing the formation and stability of synapses (Kang et al., 2015).

Previous studies have shown that AP supports pro-active metabolism and improves pro-active immunity in mammals (Dorsher and McIntosh, 2011). EA at the “Zusanli” point (ST36) and “Neiting” point (ST44) promotes microcirculation and neuronal integrity in the spinal cord of adult rats (Jiang et al., 2015). In view of these findings, it deemed important to gain more insights into molecular mechanisms underlying the beneficial functions of EA at Jiaji points that contribute to recovery. L1 has been shown to be a likely contributor to improvement of functions after SCI (Guseva et al., 2014; for a recent review see Sytnyk et al., 2017). We have previously demonstrated that EA is mediated, at least in part, by promotion of L1 expression and by differentially modulating the expression levels of GFAP and nestin at distinct time points after SCI and that EA is more effective than AP alone in promoting locomotor recovery (Wei et al., 2017). Previous studies demonstrated that L1 signals through the chain of src, fyn and Erk by phosphorylation and that L1 reduces levels of PTEN and p53 (Wang et al., 2012; Dou et al., 2013; Sytnyk et al., 2017). Erk activation mediates L1-stimulated neurite outgrowth through src. Phosphorylation of Erk1/2 protects nerve cells (Zeng et al., 2017). Activation of Erk1/2 induces apoptosis and the S-phase of inflammatory responses in the spinal cord after SCI, being considered to promote the restoration of the damaged spinal cord function after SCI (Maness and Schachner, 2007; Wang et al., 2016; Zhu et al., 2016). Indeed, we now found that EA enhances pErk levels after SCI earlier than AP, showing the effectiveness of EA in recovery after SCI.

In addition, we show that EA influences activities of Akt, Bad and caspases, which mediate neuritogenesis and neuroprotection and are specifically and differentially triggered by activation of L1 (Kilic et al., 2017). Furthermore, we have shown that L1 is associated with CK2 and that deficiency PTEN and p53 promotes neuritogenesis *in vitro* and regeneration after trauma (Wang and Schachner, 2015). PTEN and mTOR represent a signaling pathway that directly influences dendritic sprouting, axonal plasticity, and regeneration (Huang et al., 2017; Kwon et al., 2017). Enhanced mTOR activity reduces death of motor neurons, protects the damaged nerve tissue, reduces formation of syringomyelia after SCI and contributes to repair (Wang et al., 2015). We now show that EA promotes the phosphorylation of mTOR and inhibits the expression of PTEN and p53. EA inhibits the expression of p53 and Noxa in the hippocampus of the rat vascular dementia model and increases resistance to apoptosis. The combined observations extend our knowledge on the molecular mechanisms underlying the beneficial effects of AP and EA in the context of L1 functions. However, further studies are needed to more extensively understand the molecular mechanisms underlying changes of L1-dependent signaling molecules analyzed in the present study. Our study suggests a complex network of interdependencies of mechanisms. To evaluate these interdependencies will be interesting, but would require a very extensive study, requiring an elaborate dissection of each of the parameters that were here investigated. If performed systematically this dissection would go beyond the scope of the present study. Also interesting would be to find links to the functions of other signaling molecules in the context of L1 functions, remaining to be investigated in the future with the hope that these will contribute to substantiating the beneficial roles of AP and EA in recovery after SCI.

## Author Contributions

MS and ZW designed the experiments. ZW performed the experiments and analyzed the data together with MS. ZW wrote a draft of the article. WZ and MS modified the draft. MS prepared the version for submission.

## Conflict of Interest Statement

The authors declare that the research was conducted in the absence of any commercial or financial relationships that could be construed as a potential conflict of interest.
